# A new nomogram based on ultrasound and clinical features for distinguishing epididymal tuberculosis and nontuberculous epididymitis

**DOI:** 10.1038/s41598-024-65682-1

**Published:** 2024-07-02

**Authors:** Pengju Liu, Hai Gu, Guofeng Cai, Yong Qin

**Affiliations:** Department of Urology, Zhejiang Chinese Medicine and Western Medicine Integrated Hospital, 208 East Huancheng Road, Hangzhou, China

**Keywords:** Epididymal tuberculosis, Diagnostic model, Ultrasound examination, Nontuberculous epididymitis, Tuberculosis, Urinary tract infection

## Abstract

Using ultrasound findings and clinical characteristics, we constructed and validated a new nomogram for distinguishing epididymal tuberculosis from nontuberculous epididymitis, both of which share similar symptoms. We retrospectively examined data of patients with epididymal tuberculosis and nontuberculous epididymitis hospitalized between January 1, 2013, and March 31, 2023. Eligible patients were randomly assigned to derivation and validation cohorts (ratio, 7:3). We drew a nomogram to construct a diagnostic model through multivariate logistic regression and visualize the model. We used concordance index, calibration plots, and decision curve analysis to assess the discrimination, calibration, and clinical usefulness of the nomogram, respectively. In this study, 136 participants had epididymal tuberculosis and 79 had nontuberculous epididymitis. Five variables—C-reactive protein level, elevated scrotal skin temperature, nodular lesion, chronic infection, and scrotal skin ulceration—were significant and used to construct the nomogram. Concordance indices of the derivation and validation cohorts were 0.95 and 0.96, respectively (95% confidence intervals, 0.91–0.98 and 0.92–1.00, respectively). Decision curve analysis of this nomogram revealed that it helped differentiate epididymal tuberculosis from nontuberculous epididymitis. This nomogram may help clinicians distinguish between epididymal tuberculosis and nontuberculous epididymitis, thereby increasing diagnosis accuracy.

## Introduction

Tuberculosis (TB) is a disease caused by *Mycobacterium tuberculosis.* In 2021, ~ 10 million new TB cases and 1.6 million TB-related deaths were reported; these numbers were higher than those reported in 2020^1^. Thus, TB continues to be a serious threat to public health. Although it usually affects the lungs, it can also affect other organs^2^. Genitourinary TB accounts for ~ 27% extrapulmonary TB cases^3^. Epididymal tuberculosis (ETB) is the most common form of male genital TB and is often secondary to renal TB; isolated ETB is rare^4^. Because the clinical symptoms of ETB are nonspecific, early diagnosis is challenging. ETB is often misdiagnosed as nontuberculous epididymitis, consequently delaying correct treatment. Therefore, distinguishing between ETB and nontuberculous epididymitis is crucial for appropriate treatment^5^. Ultrasound examination is commonly used in clinical examination to diagnose ETB. Its advantages include simplicity of use, noninvasiveness, and freedom from radiation^6^. This study aimed to use ultrasound findings and clinical characteristics to construct a new nomogram to distinguish between ETB and nontuberculous epididymitis at an early stage, which can help improve ETB diagnostic accuracy.

## Methods

### Study design and patients

In this retrospective study, we assessed the records of consecutive patients with ETB and nontuberculous epididymitis at our hospital from January 1, 2013 to March 31, 2023. The inclusion criteria were age of > 18 years and confirmation of ETB diagnosis with one of the following criteria: histopathological diagnosis, positive *M. tuberculosis* culture (sinus tract discharge or pus), and effectiveness of anti-TB treatment (Improvement of clinical symptoms in patients). Nontuberculous epididymitis diagnosis was confirmed by one of the following criteria: effectiveness of antibiotic treatment (Improvement of clinical symptoms in patients), positive results of bacterial culture (pus), and histopathological diagnosis of nontuberculous epididymitis. Patients < 18 years age or those positive for HIV were excluded from this study. We assessed common ultrasound findings and clinical symptoms in this study alongside certain demographic characteristics and laboratory test results. All ultrasound examinations were completed in our hospital. Each patient who met the inclusion criteria of the study was randomly assigned to one of two cohorts: derivation (70%) or validation (30%). Data from the derivation cohort were used to identify pertinent variables and construct a diagnostic model and those from the validation cohort were used to validate the nomogram that depicted the model.

### Definition of variables

The following variables were initially included in this study: age, diabetes, previous TB history, lower urinary tract symptoms (LUTS), fever, night sweats, scrotal mass, elevated scrotal skin temperature, scrotal skin ulceration, lesion location, chronic infection, heterogeneously hypoechoic lesion, nodular lesion, thickened scrotal skin, hydrocele, intrascrotal extratesticular calcification, scrotal abscesses, and scrotal sinus tract, erythrocyte sedimentation rate (ESR), C-reactive protein (CRP), purified protein derivative (PPD)result, and urine leukocyte count. Herein, diabetes was defined according to the guidelines proposed by the World Health Organization^7^. LUTS primarily included frequent urination, urgency, and pain during urination. Furthermore, fever was defined as a body temperature exceeding 37.5℃. Heterogeneously hypoechoic lesion, nodular lesion, thickened scrotal skin, hydrocele, intrascrotal extratesticular calcification, scrotal abscesses, and scrotal sinus tract were examined by reviewing the patient’s ultrasound report in our hospital. Ultrasound manifestations of nodular lesions was described as follows: clear boundaries between the lesion tissue and normal tissue, heterogeneously hypoechoic, and presence of calcifications. Chronic infection was defined as a disease that lasted for > 3 months. A positive PPD result was considered to represent a scleroma lesion with a diameter of > 5 mm. A urine leukocyte result was considered positive if white blood cell levels in routine urine tests were elevated.

### Data analysis and model construction

Herein, data for categorical variables were calculated as frequencies and percentages (%) and those for continuous variables were calculated as means ± standard deviations or as medians and interquartile ranges, depending on the data distribution. To compare the data of patients with ETB and those with nontuberculous epididymitis, we used unpaired *t*–test, Wilcoxon rank–sum test, Pearson chi-square test, or Fisher’s exact test, as appropriate. A *P* level of < 0.05 was considered statistically significant. We used R version 4.1.2 (The R Foundation, Vienna, Austria) for statistical analysis.

The diagnostic model was constructed via the following steps: first, we performed univariable analysis of 22 variables; those for which *P* < 0.2 were used in further multivariable analysis. Variables whose *P* level was < 0.05 in the multivariable analysis were selected to construct the model. Second, we calculated the odds ratio for each variable through multivariable logistic regression analysis and constructed the diagnostic model. To visualize the model, we drew a nomogram. Third, we validated the diagnostic model through the derivation and validation cohorts. We evaluated the discrimination (concordance index) and calibration (calibration plots and *P* value in the Hosmer–Lemeshow test) of the diagnostic model, and the nomogram was subjected to decision curve analysis.

### Ethics approval and consent to participate

This study complied with the declaration of Helsinki and was approved by the Human Research Ethics Committee of Zhejiang Chinese Medicine and Western Medicine Integrated Hospital (Approval No: 2023–141). Informed consent was waived owing to the retrospective nature of the study and approved by the Human Research Ethics Committee of Zhejiang Chinese Medicine and Western Medicine Integrated Hospital.

## Results

Through screening, 215 eligible patients were ultimately included in this study. Of these patients, 136 had ETB (81 were diagnosed through histopathology, 25 were diagnosed through MTB culture, and 30 were diagnosed through diagnostic anti-tuberculosis treatment) and 79 had nontuberculous epididymitis (21 were diagnosed through histopathology, 1 was diagnosed through bacterial culture, and 57 were diagnosed through effective antibacterial treatment). The patients’ demographic and other characteristics are presented in Table 1. We randomly assigned patients to derivation and validation cohorts in a 7:3 ratio; the derivation cohort included 96 patients with ETB and 56 with nontuberculous epididymitis and the validation cohort included 40 with ETB and 23 with nontuberculous epididymitis. We used the data of the derivation cohort for constructing the diagnostic model.

**Table 1:** Baseline characteristics of the patients and the results of univariable analysis.

Characteristic	Patients (N = 215)		*P* value
	ETB (n = 136)	Nontuberculous epididymitis (n = 79)	
Median age, years	47 (IQR, 33.25–60)	56 (IQR, 35.5–65.5)	.01
Diabetes, n			< .11
No	128 (94%)	66 (84%)	
Yes	8 (6%)	13 (16%)	
Previous TB history, n			< .01
No	69 (51%)	72 (91%)	
Yes	67 (49%)	7 (9%)	
Scrotal mass, n			< .01
No	80 (59%)	73 (92%)	
Yes	56 (41%)	6 (8%)	
Scrotal skin ulceration, n			< .01
No	80 (59%)	76 (96%)	
Yes	56 (41%)	3 (4%)	
Elevated scrotal skin temperature, n			< .01
No	129 (95%)	42 (53%)	
Yes	7 (5%)	37 (47%)	
Lesion location, n			.41
Unilateral	94 (69%)	48 (61%)	
Bilateral	42 (31%)	31 (39%)	
LUTS, n			< .01
No	103 (76%)	29 (37%)	
Yes	33 (24%)	50 (63%)	
Fever, n			< .01
No	123 (90%)	49 (62%)	
Yes	13 (10%)	30 (38%)	
Night sweats, n			.04
No	113 (83%)	75 (95%)	
Yes	23 (17%)	4 (5%)	
Heterogeneously hypoechoic lesion, n			.05
No	16 (12%)	19 (24%)	
Yes	120 (88%)	60 (76%)	
Nodular lesion, n			.01
No	101 (74%)	76 (96%)	
Yes	35 (26%)	3 (4%)	
Thickened scrotal skin, n			< .01
No	96 (71%)	75 (95%)	
Yes	40 (29%)	4 (5%)	
Hydrocele, n			.05
No	104 (76%)	52 (66%)	
Yes	32 (24%)	27 (34%)	
Intrascrotal calcification, n			.48
No	130 (96%)	77 (97%)	
Yes	6 (4%)	2 (3%)	
Scrotal abscesses, n			.98
No	103 (76%)	78 (99%)	
Yes	33 (24%)	1 (1%)	
Scrotal sinus tract, n			.01
No	107 (79%)	77 (97%)	
Yes	29 (21%)	2 (3%)	
Median ESR (mm/h)	20.5 (IQR, 10–32.25)	25 (IQR, 14–36.5)	.24
Median CRP (mg/L)	7.88 (IQR, 3.67–13.54)	32.01 (IQR, 12.02–55.68)	< .01
Urine leukocyte, n			.02
No	69 (51%)	22 (28%)	
Yes	67 (49%)	57 (72%)	
PPD, n			< .01
Negative	70 (51%)	76 (96%)	
Positive	66 (49%)	3 (4%)	
Chronic infection, n			< .01
No	45 (33%)	71 (90%)	
Yes	91 (67%)	8 (10%)	

CRP, C-reactive protein; ESR, erythrocyte sedimentation rat; ETB, epididymal tuberculosis; IQR, interquartile range; LUTS, lower urinary tract symptoms; PPD, purified protein derivative; TB, tuberculosis.

The following variables, which had a *P* level of < 0.05 in the multivariable analysis, were ultimately used to construct the diagnostic model: CRP level, elevated scrotal skin temperature, nodular lesion, chronic infection, and scrotal skin ulceration. Multivariable logistic regression analysis was based on these variables. The odds ratio for each variable is shown in Table 2.

**Table 2:** Results of multivariable logistic regression analysis.

Intercept and variable	β	Odds ratios (95% CI)	*P* value
Intercept	0.65		.15
CRP	$-$ 0.05	0.95 (0.91–0.98)	< .01
Elevated scrotal skin temperature	$-$ 3.27	0.04 (0.01–0.17)	< .01
Nodular lesion	4.67	105.70 (7.08–3775.61)	< .01
Chronic infection	2.23	9.27 (2.85–37.20)	< .01
Scrotal skin ulceration	3.34	28.34 (4.63–313.55)	< .01
Concordance index			
Derivation cohort	0.95 (0.91–0.98)		
Validation cohort	0.96 (0.92–1.00)		

CI, confidence interval; CRP, C-reactive protein.

**Figure 1:**
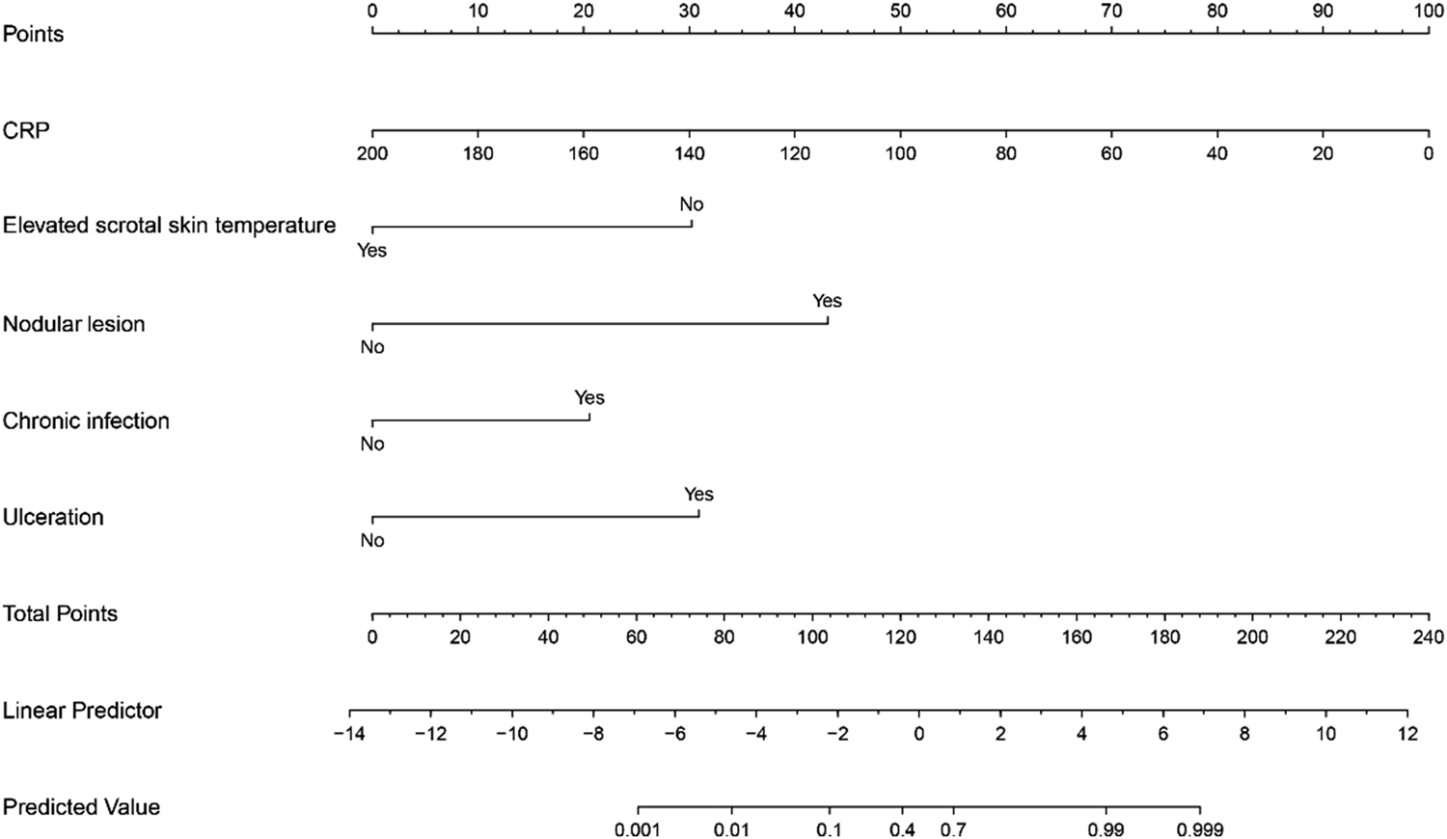
Nomogram for differentiating between epididymal tuberculosis and nontuberculous epididymitis. “Chronic infection” refers to disease of > 3 months’ duration; “ulceration” refers to scrotal skin ulceration. Abbreviation: CRP, C-reactive protein.

**Figure 2:**
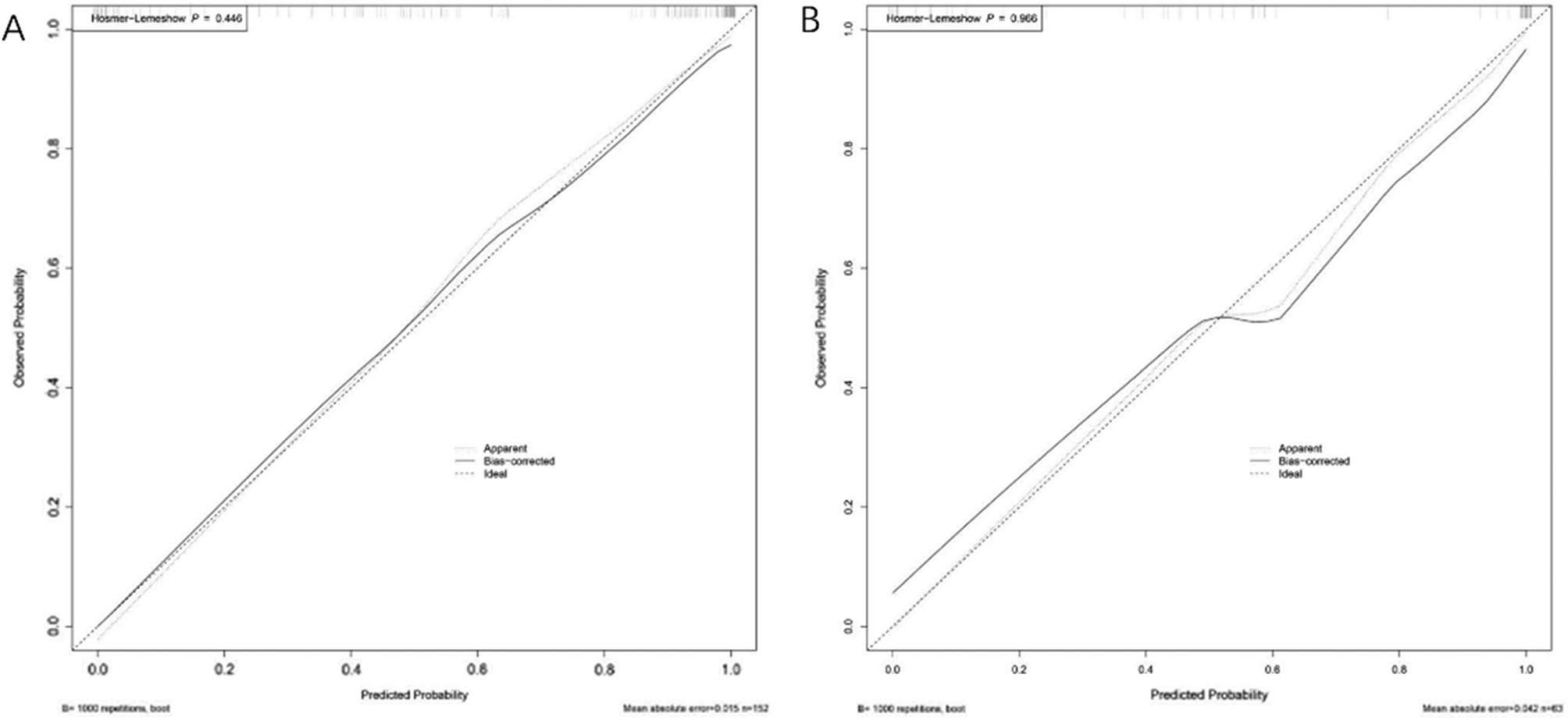
Calibration curves of the nomogram. (**A**) Derivation cohort. (**B**) Validation cohort. The significant result of the Hosmer–Lemeshow test (*P* > .05) suggested that the nomogram has goodness of fit.

We drew a nomogram to visualize the diagnostic model (Fig. 1). The ability of the nomogram to distinguish the two diseases was validated for the derivation and validation cohorts. When the model was validated for the derivation cohort, the concordance index was 0.95 (95% confidence interval [CI], 0.91–0.98). When the model was validated for the validation cohort, the concordance index was 0.96 (95% CI, 0.92–1.00). The model was well calibrated for the derivation and validation cohorts. The Hosmer–Lemeshow test yielded *P* values of 0.45 and 0.97 for the derivation and validation cohorts, respectively (Fig. 2), indicating a perfect fit between the model’s predicted values and the observed values.

**Figure 3:**
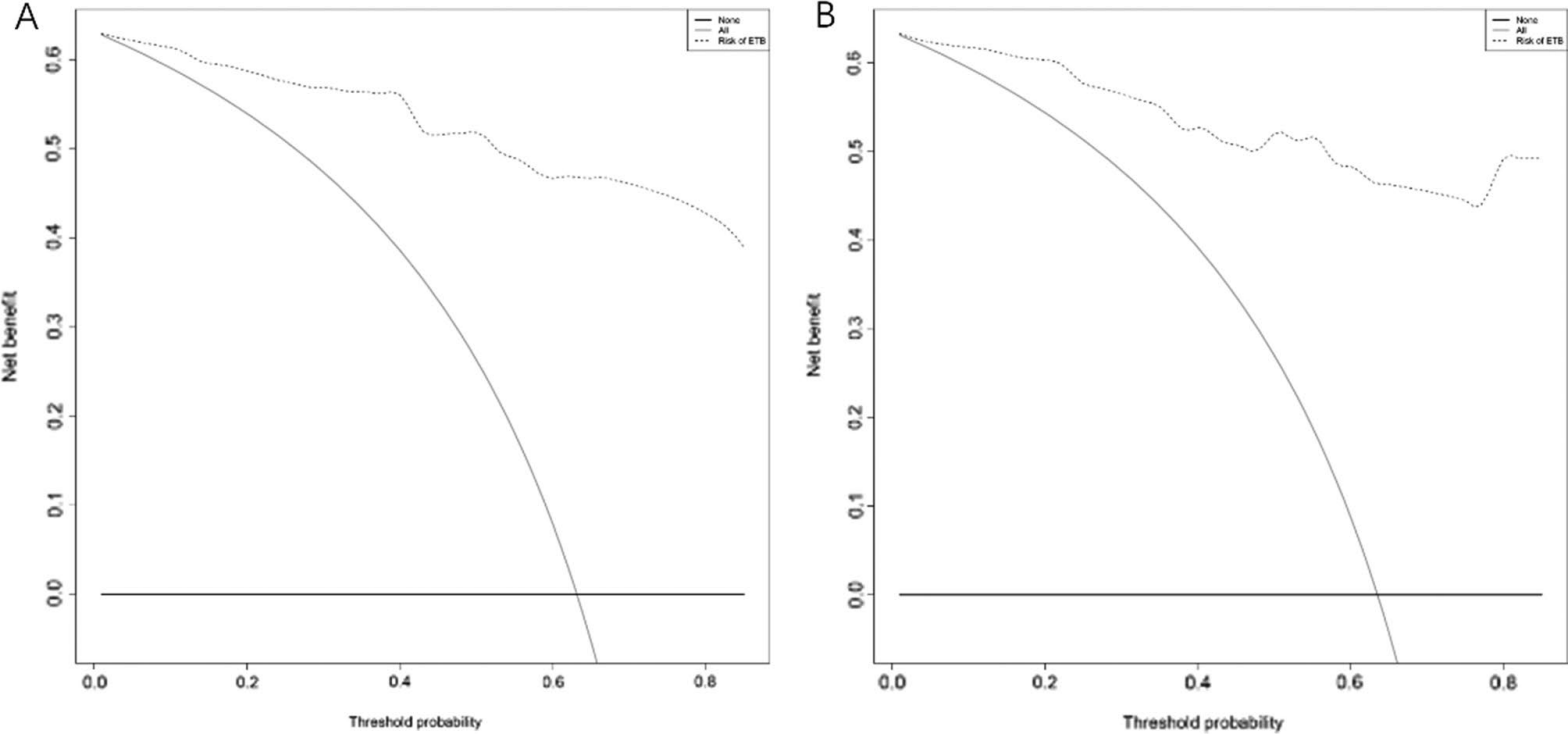
Results of the decision curve analysis of the nomogram. (**A**) Derivation cohort. (**B**) Validation cohort.

We conducted a decision curve analysis to verify the clinical application value of this diagnostic model. The result revealed that using this model to differentiate between ETB and nontuberculous epididymitis produced a net benefit for almost all threshold probabilities in the derivation and validation cohorts (Fig. 3).

## Discussion

TB remains a serious threat to global public health^8^ and is one of the most common infectious diseases and major fatal infectious diseases, especially among people living with human immunodeficiency virus^9,10^. The disease is caused by *M. tuberculosis*^11^. Pulmonary TB is the most common type of TB; however, TB often affects other organs, such as the kidneys and brain^12^. A retrospective study has revealed that ~ 33% 19,279 hospitalized patients with pulmonary TB in China had extrapulmonary TB^13^. Urogenital TB is one of the most common extrapulmonary manifestations^14–16^, and ETB is the most common male genital form of TB. ETB is observed in 7% of all TB cases^17^.

ETB is often secondary to the hematogenous dissemination of a distant primary focus. The involvement is usually unilateral however, in 25% TB cases, it may be bilateral^6^. ETB can result from the retrograde canalicular descent of *M. tuberculosis* from the prostate through the vas deferens^18,19^. Because the clinical symptoms of ETB are nonspecific, early ETB diagnosis is challenging. In fact, delay in diagnosis may endanger the patient’s physical and mental health. Lack of awareness regarding ETB may result in serious consequences such as infertility and sexual dysfunction in patients. ETB diagnosis is usually confirmed by the isolation and culturing of *M. tuberculosis* from the epididymis. However, *M. tuberculosis* culturing takes a long time—the Löwenstein–Jensen solid culture method takes 4–6 weeks—and is not conducive to rapid diagnosis. However, molecular biological diagnostic technology has also been used in diagnosing ETB^20,21^. Applying this technology can accelerate ETB diagnosis. However, obtaining test specimens is one of the difficulties in diagnosing extrapulmonary TB.

The diagnosis of numerous forms of extrapulmonary TB requires invasive diagnostic sampling^22^. In some previous studies, researchers have used fine-needle aspiration and core needle biopsy to collect samples for diagnosing ETB^23,24^. Because invasive diagnostic techniques pose potential risks to patient health; however, their clinical application is limited.

According to one report, physicians’ lack of awareness regarding testicular TB and ETB led to diagnosis via unnecessary surgery in 69% patients with these diseases^25^. Imaging studies, especially ultrasound examination, also play an important role in diagnosing ETB^26^. The ultrasound manifestations of ETB include lesions that are diffusely enlarged and heterogeneously or homogeneously hypoechoic as well as nodular. Other associated ultrasound manifestations include thickened scrotal skin, hydrocele, calcification, scrotal abscesses, and scrotal sinus tract^27^. Kim et al.^5^ believed that enlarged heterogeneous lesions of the epididymis differentiate ETB from nontuberculous epididymitis. The advantages of ultrasound examination include its noninvasiveness, simplicity of performance, and high-resolution and real-time image production. Owing to its low cost, it is especially suitable for use in underdeveloped regions. However, distinguishing ETB from nontuberculous infection via ultrasound examination is difficult^26^ because the two diseases have several ultrasound imaging similarities; therefore, ultrasound findings must be evaluated along with clinical manifestations and experimental examination results. Our diagnostic model, which is based on ultrasound manifestations and clinical characteristics, can help clinicians distinguish between ETB and nontuberculous infections to establish the diagnosis.

Herein, we initially evaluated 22 variables, of which 5—CRP level, elevated scrotal skin temperature, nodular lesion, chronic infection, and scrotal skin ulceration—were identified via logistic regression as significant and were used to construct the diagnostic model. To visualize this model, we constructed a nomogram. In validating the nomogram using the derivation and validation cohorts, we found that the model has good discrimination and calibration. Finally, to explore the clinical application value of this model, we performed decision curve analysis, which revealed that this nomogram differentiated between ETB and nontuberculous epididymitis. Because the diagnostic model accounts for laboratory examination methods and ultrasound examinations, the nomogram can be used in primary care hospitals, and because it is inexpensive, it can be used easily in underdeveloped areas.

This study had several limitations. First, some patients with epididymal tuberculosis may have received treatment with fluroquinolones and aminoglycosides prior to diagnosis, which may lead to changes in the patient’s clinical symptoms or laboratory test results. Second, the number of patients included in this study was limited. Third, external data could not be validated. Therefore, we believe that further multicenter prospective research with our diagnostic model and nomogram is needed. Moreover, including more variables in the future will be beneficial for constructing a more accurate predictive model.

## Conclusions

Herein, we constructed and validated a diagnostic model to differentiate between ETB and nontuberculous epididymitis according to the patient’s ultrasound manifestations and clinical characteristics. We visualized the model by drawing a nomogram, which incorporated ultrasound and clinical characteristics of patients. In validating the diagnostic model, we found that it has good discrimination and calibration. Decision curve analysis also revealed that the proposed nomogram could be used to differentiate between ETB and nontuberculous epididymitis. This nomogram may help clinicians in diagnosing ETB and nontuberculous epididymitis.

## Data Availability

Datasets used and analyzed during the current study are available from the corresponding author on reasonable request.
